# Preparation of polyphosphazenes: a tutorial review

**DOI:** 10.1039/c6cs00340k

**Published:** 2016-06-17

**Authors:** Sandra Rothemund, Ian Teasdale

**Affiliations:** a NanoScience Technology Center , University of Central Florida , 12424 Research Parkway Suite 400 , Orlando , FL 32826 , USA; b Institute of Polymer Chemistry , Johannes Kepler University , Altenberger Strasse 69 , 4040 Linz , Austria . Email: Ian.teasdale@jku.at

## Abstract

Poly(organo)phosphazenes are a family of inorganic molecular hybrid polymers with very diverse properties due to the vast array of organic substituents possible. This tutorial review aims to introduce the basics of the synthetic chemistry of polyphosphazenes, detailing for readers outside the field the essential knowledge required to design and prepare polyphosphazenes with desired properties. A particular focus is given to some of the recent advances in their chemical synthesis which allows not only the preparation of polyphosphazenes with controlled molecular weights and polydispersities, but also novel branched architectures and block copolymers. We also discuss the preparation of supramolecular structures, bioconjugates and *in situ* forming gels from this diverse family of functional materials. This tutorial review aims to equip the reader to prepare defined polyphosphazenes with unique property combinations and in doing so we hope to stimulate further research and yet more innovative applications for these highly interesting multifaceted materials.

## Abstract 2

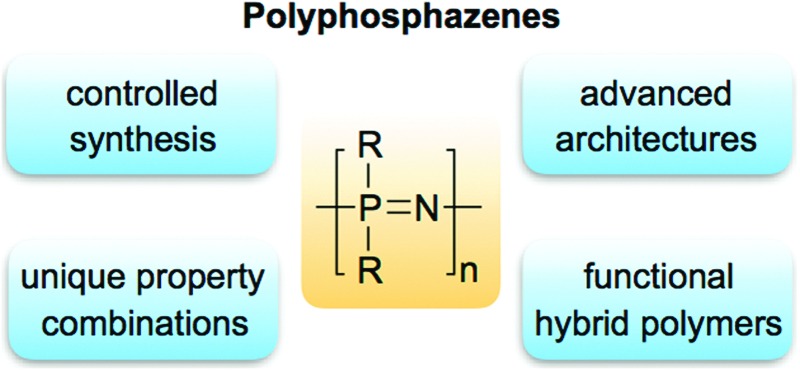
The essentials of the synthetic chemistry of poly(organo)phosphazenes are detailed in this tutorial review, with a particular focus on the recent advances in this field.

Key learning points

(1) Poly(organo)phosphazenes are a family of inorganic molecular hybrid polymers based on a phosphorus–nitrogen backbone substituted with organic side groups which show very differing properties due to the vast array of organic substituents possible.

(2) Although the breadth of properties observed can be bewildering, the main motivation for synthesising polyphosphazenes is a small number of unique combinations of properties which make polyphosphazenes extremely interesting materials.

(3) The basic synthesis, often thought to be comparatively tricky, has for a long time hindered development, herein we aim to equip the reader with the tools to design and prepare polyphosphazenes with desired properties.

(4) In this review we also describe how recently improved chemistry allows for controlled synthesis of functional polyphosphazenes with well-defined architectures such as block copolymers and branched architectures.

## 1. Introduction

### 1.1 Background

**Fig. 1:**
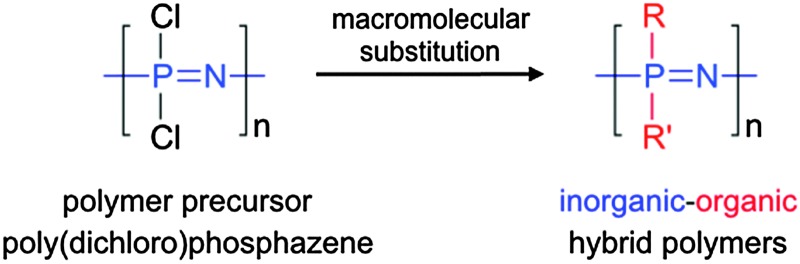
Generic structure of poly(organo)phosphazenes, a family of molecular hybrid polymers, most-commonly prepared from the precursor poly(dichloro)phosphazene [NPCl_2_]_*n*_.

**Fig. 2:**
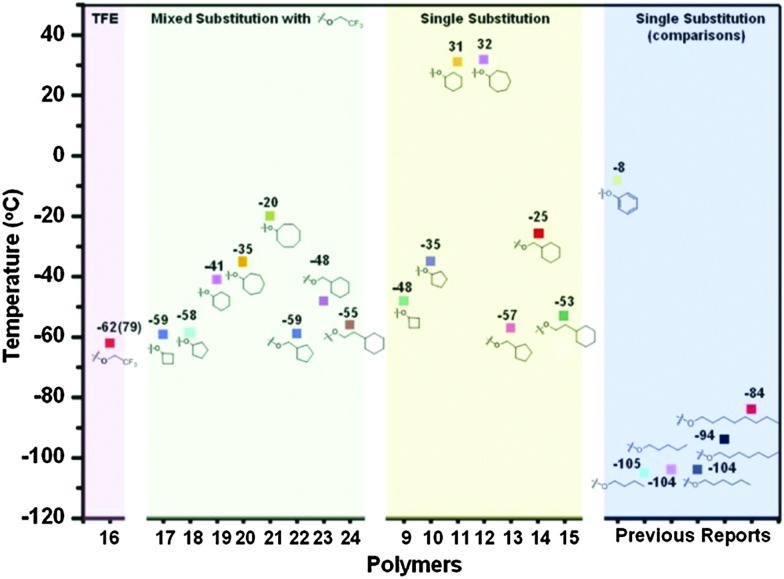
The effect of different substituents on the glass transition temperatures of poly(organo)phosphazenes, exemplifying how small structural changes can be used to tailor the physical properties of the resulting polyphosphazene. Reprinted with permission from Z. Tian *et al.*, *Macromolecules*, 2015, **48**, 4301. Copyright 2015 American Chemical Society.^2^

Polyphosphazenes are macromolecules with a phosphorus–nitrogen backbone, substituted by two (commonly organic) side groups on the phosphorus atoms to give poly(organo)phosphazenes (Fig. 1). Polyphosphazenes themselves have a long history with cross-linked elastomeric materials (‘inorganic rubber’) consisting of phosphorus and nitrogen being reported as early as the 1890's. The isolation of soluble poly(dichloro)phosphazene [NPCl_2_]_*n*_, which had formed the basis of such ‘inorganic rubbers’, was first achieved in the 1960's by H. R. Allcock and coworkers.^1^ Key to this development was the insightful observation that the cross-linking of ‘inorganic rubber’ was caused by reaction of [NPCl_2_]_*n*_ with trace amounts of H_2_O and thus, in the absolute absence of atmospheric moisture, non-cross-linked and soluble macromolecules of [NPCl_2_]_*n*_ could be prepared. As was appreciated by H. R. Allcock *et al.*, the reaction of H_2_O with [NPCl_2_]_*n*_ was *via* nucleophilic attack and therefore the chlorine atoms of [NPCl_2_]_*n*_ can also be readily replaced by organic nucleophiles to give stable poly(organo)phosphazenes, which can be processed and formed into materials with a wide variety of properties.^1^ Added to the fact that mixed substitution is also possible, *i.e.* the substitution of two or more organic substituents on the same macromolecule, this gives ready access to extensive libraries of polymers with varied properties. Indeed, small changes in the substituents can lead to considerable changes in the polymer properties, *e.g.* glass transition temperatures, which can vary from –100 °C to above room temperature (up to +300 °C are reported), with very minor structural changes (Fig. 2).

This ease of tunability of poly(organo)phosphazenes, combined with the distinctive inorganic backbone, has led to significant academic interest and the development of materials with very differing characteristics, ranging from superhydrophobic to water soluble and from cationic to anionic. Such materials have been investigated for applications as far afield as vaccine delivery^3^ and fuel cell membranes.^4^ Currently, there is significant ongoing development of polyphosphazenes for potential use in the biomedical field, including as matrices for tissue engineering^5^ as well as drug and gene delivery,^6^ primarily due to their inherent backbone degradability and biocompatibility.^7^ Polyphosphazenes are further used in a variety of functional materials, for example as high performance elastomers,^8^ due to their combination of chain flexibility and high temperature stability, or as flame retardants^9^ due to their side chain functionality and high limiting oxygen indices. For a detailed overview on the application of polyphosphazenes as soft materials, the reader is referred to a review article recently published elsewhere.^8^ Furthermore, a detailed and highly comprehensive review of the field, including a summary of the majority of the poly(organo)phosphazenes produced up to 2003, has been published by H. R. Allcock^1^ and thus the reader is referred here for further information on the historical works. The focus of this review is laid on more recent developments and above all in synthetic methods for the preparation of polyphosphazene based materials.

### 1.2 Molecular level hybrids

Poly(organo)phosphazenes can be thought of as ‘molecular level hybrids’ with the effects of the organic substituents dictating the properties of the inorganic backbone and *vice versa*. The organic component has a defining role in terms of the chemical properties and thus the solubility and functionality of the resulting polymer, as well as hydrolytic stability (hence degradability). The inorganic backbone itself offers unique properties and is clearly more than just an inert scaffold, determining the size, architecture and conformation of any hybrid polymer formed. It is important to note that the P–N backbone has an unusual form of bonding, almost unique in chemistry and a detailed understanding of the bonding remains elusive.^10^ Due to its unique skeletal-bonding structure, it is not unsaturated, quite different from electron rich organic systems, nor is it delocalised. Indeed, far from being conjugated, the free rotation around the P–N bonds is extremely low which translates into the experimental observations that the linear phosphazene backbone can be quite flexible (clearly depending on the nature of the organic substituents).

## 2. Synthesis of polyphosphazenes

### 2.1 Macromolecular substitution of [NPCl_2_]_*n*_

**Fig. 3:**
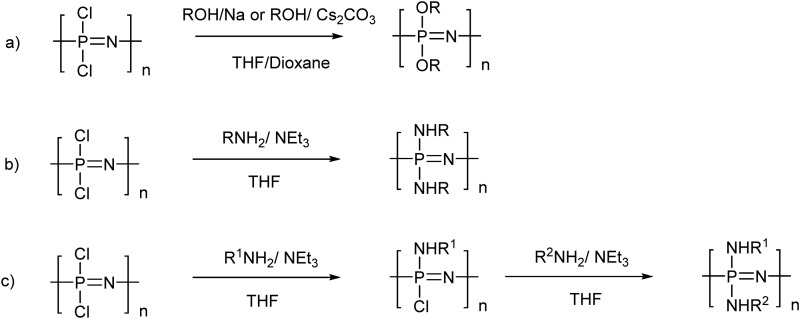
The most common routes to poly(organo)phosphazenes *via* macromolecular substitution of the reactive precursor poly(dichloro)phosphazene using alkoxides or aryloxides (a) and primary amines (b). Scheme (c) depicts a mixed substitution using different nucleophiles which allows fine-tuning the properties of the polymer.

Although not exclusive, by far the most widely used route to poly(organo)phosphazenes is *via* the precursor poly(dichloro)phosphazene [NPCl_2_]_*n*_ (Fig. 3). Due to its high reactivity, this intermediate can only be isolated under anhydrous conditions and must be carefully stored or immediately reacted further. Recently, however, significant progress has been made in this regard, with the discovery that [NPCl_2_]_*n*_ can be readily stored in solution in the presence of diglyme.^11^ Although hitherto unclear how, the remarkable stabilisation effect enables storage of [NPCl_2_]_*n*_ in solution for several years, whereby previously hydrolysis and/or cross-linking would have been expected within hours.

**Fig. 4:**
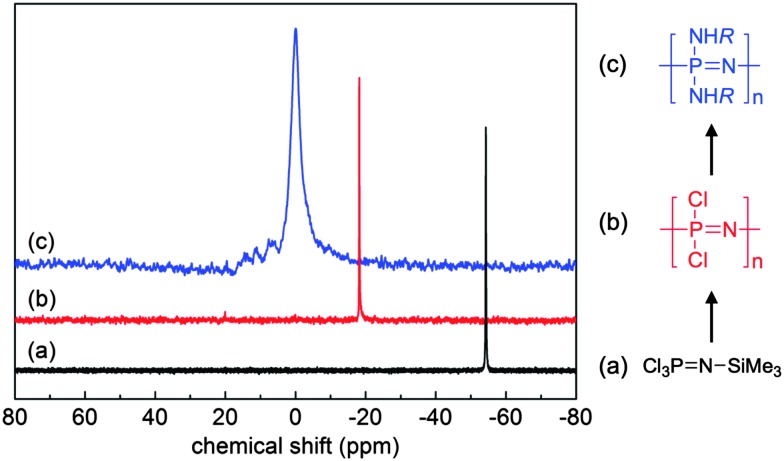
Sample ^31^P{^1^H} NMR spectra of the monomer trichlorophosphoranimine Cl_3_PNSiMe_3_ (a), polydichlorophosphazene [NPCl_2_]_*n*_ (b) and a macrosubstituted poly(organo)phosphazene [NP(NHR)_2_]_*n*_ (c) demonstrating the simple tracking of the reaction progression. Adapted from H. Henke *et al.*, *J. Polym. Sci., Part A: Polym. Chem.*, 2013, **51**, 4467. Copyright 2013 Wiley Periodicals, Inc.^12^

In the absence of H_2_O, the high reactivity of [NPCl_2_]_*n*_ in turn allows complete post-polymerisation with mono-functional nucleophiles, assuming functional group tolerance (*e.g.* no free acid groups). This facile post-polymerisation substitution of [NPCl_2_]_*n*_ to produce the final poly(organo)phosphazenes has been coined “macromolecular substitution”^1^ and although enabling the simple preparation of a wide range of polymers from a single precursor, such a macromolecular reaction should not be underestimated since large numbers of simultaneous reactions on a single macromolecule are required and reaction conditions and times can greatly vary depending on the nucleophilicity and steric hindrance of the substituent.^2^ Furthermore, it is important to ensure complete replacement of Cl atoms, not only for the sake of purity, but also due to the high susceptibility of the P–Cl bond to hydrolysis and the subsequent uncontrolled cross-linking and/or degradation that may occur should any unreacted Cl groups be left remaining on the final polymer. ^31^P{^1^H} NMR spectroscopy allows relatively simple tracking of the macromolecular substitution reaction (Fig. 4b and c), whereby a single peak corresponding to [NPCl_2_]_*n*_ at –18 ppm should completely shift to the substituted polymer (although caution should be exercised due to the limits in sensitivity of NMR spectroscopy and the possibility of impurities in which the backbone phosphorus is monosubstituted).^2^

Mixed substitution, usually *via* stepwise addition of different nucleophiles, gives simple access to an even broader range of materials, what are essentially random copolymers (Fig. 3c). When conducting such substitution reactions, one should be aware that substituent exchange reactions can also occur, thus R^1^ may be displaced by R^2^ to some extent, depending on the nucleophilicity and steric hindrance of the reagents.^13^

Poly(alkyl/aryl)phosphazenes [NPR_2_]_*n*_ with the organic substituent directly attached *via* a P–C bond from [NPCl_2_]_*n*_ can be synthesised *via* substitution with suitable organic nucleophiles such as RMgX or RLi. However, such reactions are often tricky due to the low functional group tolerance and thus direct methods are preferred (see Section 2.3).

### 2.2 Polymerisation methods of [NPCl_2_]_*n*_

#### 2.2.1 Ring-opening polymerisation of [NPCl_2_]_3_

**Fig. 5:**
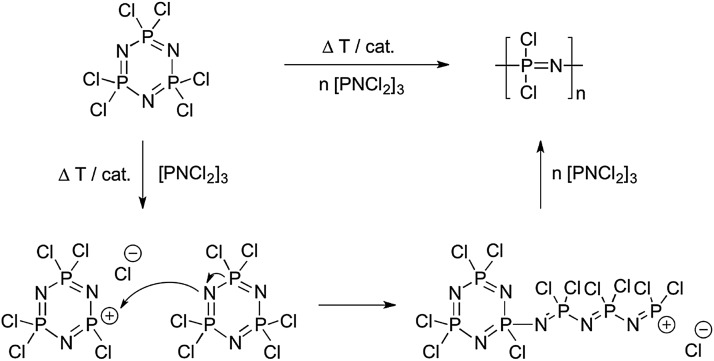
Thermally and/or catalytically induced ring opening polymerisation representing the classical route to synthesise poly(dichloro)phosphazene [NPCl_2_]_*n*_ and its commonly accepted mechanism.

Several approaches are described in the literature to synthesise poly(organo)phosphazenes, however, there are two main routes most commonly used to obtain the polymeric precursor [NPCl_2_]_*n*_. The classical route to prepare linear, high molecular weight [NPCl_2_]_*n*_ is *via* the ring-opening polymerisation of hexachlorocyclotriphosphazene [NPCl_2_]_3_ (Fig. 5). This reaction is usually carried out under vacuum in a sealed glass tube at high temperatures; typically 250 °C for several hours (see pages 146–153 in ref. 1). At temperatures around 250 °C, chlorine atoms can be cleaved from [NPCl_2_]_3_ to give a cationic phosphazenium species which initiates the ring opening of a second ring, thus propagating the polymerisation (Fig. 5). This reaction can be carried out on relatively large scales, indeed small scale <10 g reactions are often plagued with low reproducibility, presumably due to the relative effect of contaminants at lower scales or activity from the vessel surface. Efficient temperature control is essential for successful polymerisation since Cl cleavage is minimal at temperatures far below 250 °C and thus polymerisation rates are infinitively slow. Meanwhile, at temperatures above 250 °C, significant cross-linking occurs. The reaction temperature can be moderately lowered to around 200 °C by the addition of catalytic amounts of Lewis acids, most commonly anhydrous AlCl_3_. A potentially more convenient solution state method is also possible using trichlorobenzene as a solvent at 214 °C, with CaSO_4_·2H_2_O as a promoter and HSO_3_(NH_2_) as a catalyst.^14^ Furthermore, a direct synthesis from the [NPCl_2_]_3_ precursors phosphorus pentachloride (PCl_5_) and ammonium chloride has been developed,^14^ although the sublimation of PCl_5_ can become problematic at the high temperatures required for the ring-opening.

The molecular weights of the ring opening routes are usually high and difficult to control and the polydispersities are broad, primarily due to the underlying initiation mechanism (Fig. 5) in which Cl cleavage in [NPCl_2_]_3_ (and thus initiation of new polymer chains) can occur throughout the polymerisation. Furthermore, uncontrolled chain branching (especially at higher conversions) is an inherent feature of this polymerisation,^11^ due to Cl cleavage from the growing [NPCl_2_]_*n*_ chains, thus producing initiation sites along the backbone. Since this leads to cross-linked species at higher conversions, the reaction must be terminated before gelation occurs (a time point often difficult to define), leading to low yields (*ca.* 40%). Recent work showed that full conversion could be achieved *via* a catalysed room temperature ring opening polymerisation of [NPCl_2_]_3_ in 1,2-dichlorobenzene.^15^ The key to this was the use of silylium ions (SiEt_3_^+^) to form the required phosphazene cations in combination with a weakly coordinating carborane anion. Complete conversion of [NPCl_2_]_3_ to [NPCl_2_]_*n*_ was observed, and if optimised, this proof of principle would be an extremely useful and mild route to high molecular weight [NPCl_2_]_*n*_.

#### 2.2.2 Living cationic polymerisation

**Fig. 6:**
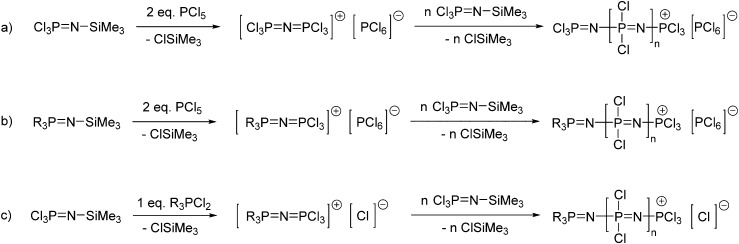
Synthesis of poly(dichloro)phosphazene [NPCl_2_]_*n*_ by living cationic polymerisation of trichlorophosphoranimine (Cl_3_PNSiMe_3_) *via* initiation with PCl_5_ (a), incorporation of alkyl or aryl substituted phosphoranimines for monodirectional growth (b) and phosphine-mediated polymerisation (c).

Controlled polymerisations have become of utmost importance in polymer chemistry, allowing access to block copolymers and more complex molecular structures and thus forming the basis of macromolecular engineering and modern polymer nanotechnology. For this purpose, defined molecular weights, narrow molecular weight distributions (*M*_w_/*M*_n_) and control over the precise nature of the polymer end groups are required, in order to be able to adapt functionality and/or to carry out further modifications. Controlled polymerisation of [NPCl_2_]_*n*_ is possible *via* a living cationic polymerisation of trichlorophosphoranimine (Cl_3_PNSiMe_3_). This can be carried out in solution at room temperature *via* reaction of Cl_3_PNSiMe_3_ with two equivalents of PCl_5_ giving a cationic species [Cl_3_PNPCl_3_]^+^ with PCl_6_^–^ as the counterion (Fig. 6a).^15^ This species can initiate the polymerisation upon addition of further equivalents of Cl_3_PNSiMe_3_ leading to polymer chains with a “living” cationic end group.^16^ One equivalent of ClSiMe_3_ is formed as a side product with every monomer molecule added to the polymer, making this polymerisation a rare example of a polycondensation reaction which occurs *via* a chain growth mechanism (compared to most polycondensation reactions having step-growth mechanisms). The living chain growth mechanism, with one cationic initiator per propagating chain, allows not only control of molecular weight *via* the feed monomer to initiator ratio, but also leads to poly(dichloro)phosphazenes with narrow polydispersities. This process can be readily followed by ^31^P NMR spectroscopy (Fig. 4a and b), confirming the consumption of Cl_3_PNSiMe_3_ and its incorporation into the polymer chain. Reaction times may vary depending on the desired polymer chain length, the monomer concentration and the nature of the counter ion,^16^ but Cl_3_PNSiMe_3_ is observed to be consumed completely relatively quickly within a few hours in the preferred solvent dichloromethane.^17^

The living cationic polymerisation requires large amounts of the monomer Cl_3_PNSiMe_3_ of high purity, usually prepared *via* reaction of PCl_3_ with LiN(SiMe_3_)_2_ to form Cl_2_PN(SiMe_3_)_2_, followed by oxidation with the chlorinating agent SO_2_Cl_2_ to give Cl_3_PNSiMe_3_. An optimized high yield synthesis route from the Manners group allows a two-step but one-pot preparation in scales of 50–100 g in yields of >80%.^18^ However, the purification of Cl_3_PNSiMe_3_ *via* vacuum distillation can still be problematic on larger, more industrial scales, and thus to circumvent this, a one-pot method to produce [NPCl_2_]_*n*_ directly *via in situ* preparation and polymerisation of Cl_3_PNSiMe_3_ has been suggested.^19^ This alternative route could facilitate upscaling and the industrial preparation of polyphosphazenes, as it avoids the vacuum distillation of the monomer. However, there is some loss in molecular weight control and thus the obtained polyphosphazenes show slightly broader polydispersities as compared to the living cationic polymerisation.

When PCl_5_ is used to form the cationic initiator for the living polymerisation of Cl_3_PNSiMe_3_ (Fig. 6a), some bidirectional growth can be observed due to the ability of the cationic propagating site to migrate.^20^ Furthermore, both end groups are identical after macrosubstitution of the chlorine atoms, thus limiting the options in terms of the preparation of polymers with higher architectures. However, monodirectional growth can be achieved *via* the use of R_3_PNSiMe_3_ type moieties (Fig. 6b).^21^ Reaction of such phosphoranimines with two equivalents of PCl_5_ gives a cationic species capable of initiating the polymerisation of Cl_3_PNSiMe_3_ *via* an identical mechanism. The R groups, typically phenyl groups, effectively block one end of the initiating species, forcing the polymerisation to proceed in only one direction and more importantly resulting in polymers with defined chain ends.

Another alternative route to polyphosphazenes with defined chain ends is provided by the initiation with chlorinated tertiary phosphines R_3_PCl_2_ (Fig. 6c). Tertiary phosphines, for example Ph_3_PCl_2_, are known to exist completely in an ionized form [Ph_3_PCl]^+^ [Cl]^–^ in polar solvents such as dichloromethane. This cationic species can be used to initiate the living cationic polymerisation of Cl_3_PNSiMe_3_^17,22^ thus enabling monodirectional growth of the polymer and leading to controlled chain growth and narrow polydispersities. Furthermore, it has been shown that the synthesis of well-defined polyphosphazenes with end group functionalities is also possible through the use of monofunctionalised tertiary phosphines, *i.e.* bearing a single functional group on one of the organic moieties.^17^ Such monofunctionalised phosphine compounds are often commercially available due to their widespread use as ligands in inorganic catalysis and thus the spectrum of functional end groups readily available is manifold.

The cationic polymerisation routes show excellent control of the molecular weight for shorter polymer chain lengths (up to approximately 50 repeat units) and narrow molecular weight distributions (*M*_w_/*M*_n_) are observed. Thereafter control decreases at higher degrees of polymerisation (DP), presumably due to the prevalence of side reactions.^17^ Furthermore, the DP's obtained *via* the cationic polymerisation routes are significantly lower than *via* the ring opening polymerisation of [NPCl_2_]_3_, whereby DP's are commonly reported to be >10 000, and as such the ring-opening method remains of importance for applications where higher molecular weights are required, but control of molecular structure is of lesser importance.

### 2.3 Direct synthesis routes to poly(organo)phosphazenes

**Fig. 7:**
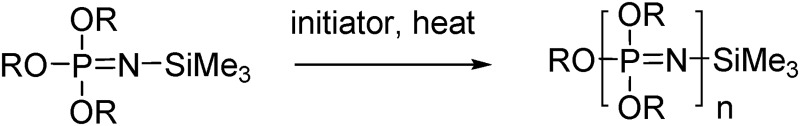
Direct synthesis route to *N*-silylphosphoranimines *via* anionic polymerisation.

It is also possible to directly synthesise poly(organo)phosphazenes, without the need for the [NPCl_2_]_*n*_ precursor. An example here includes the anionic polymerisation of *N*-silylphosphoranimines with fluoride ion initiators in the presence of *N*-methylimidazole (Fig. 7). Without quite achieving the control of the cationic routes described above, this polymerisation has living character and enables the preparation of polymers with polydispersities between 1.3 and 2.3 at 125 °C.^23^ Recently Steinke *et al.* ^24^ showed that the polymerisation of similar *N*-silylphosphoranimines can be initiated by H_2_O with a catalytic amount of *N*-methylimidazole with living polymerisation kinetics (*n* ≈ 100 and dispersity ≤1.15). This method was proven to be robust for the preparation of poly(bistrifluoroethoxy phosphazene) and thus, if extended to a wider range of monomers, this could be a valuable route to prepare poly(organo)phosphazenes with excellent control of molecular weight and polydispersity.

**Fig. 8:**
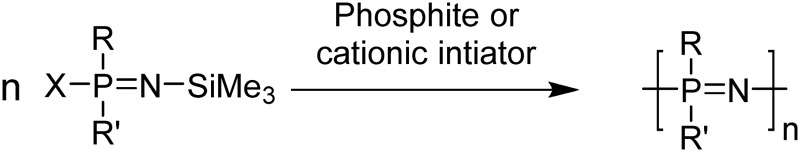
Direct synthesis route to poly(alkyl/aryl)phosphazenes, with R and R′ = alkyl, aryl, typically R = Ph and R′ = Me, X = Br or Cl.

**Fig. 9:**
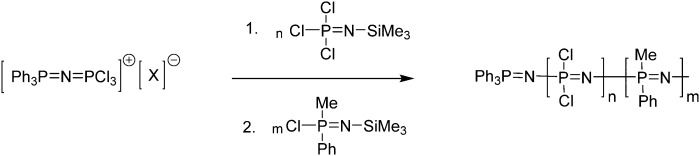
Exemplary synthesis pathway for the preparation of block copolymers with poly(dichloro)phosphazene and poly(alkyl/aryl)phosphazene.

Poly(alkyl/aryl)phosphazenes, [NPR_2_]_*n*_, whereby the organic substituent is directly attached *via* a P–C bond, can also be prepared *via* direct polymerisation routes. Initially developed by thermal (100–180 °C) condensation of (CF_3_CH_2_O)R_2_P=NSiMe_3_ type monomers,^25^ this has since been expanded to cationic initiators and with monomers of the type BrR_2_P=NSiMe_3_.^21^ Recently, the polymerisation of halo(alkyl/aryl)phosphoranimines (XR_2_PNSiMe_3_, X = Br or Cl) initiated by organic phosphites ((MeO)_3_P) has also been shown to be an effective route to poly(alkyl/aryl)phosphazenes at ambient temperature (Fig. 8).^15^ Furthermore, such phosphoranimines can be combined with the cationic polymerisation of [NPCl_2_]_*n*_ (Fig. 9), thus allowing the preparation of block copolymers as will be discussed in the next section.

### 2.4 Preparation of block copolymers

Although the macromolecular substitution of [NPCl_2_]_*n*_ gives ready access to copolymers, they are exclusively random copolymers because the nucleophilic substitution of the chlorine atoms along the polyphosphazene backbone is non-selective. Block copolymers, however, have widespread use and have proven essential for a wide number of applications, thus a considerable effort has been made in order to be able to also prepare block copolymers of polyphosphazenes. Due to living nature of the cationic polymerisation routes to [NPCl_2_]_*n*_, described above (Fig. 6), the polymer chain ends remain active and enable further polymerisation upon addition of a second monomer. Clearly, due to the macrosubstitution method, all repeat units are however then substituted to yield the same poly(organo)phosphazene. One option to circumvent this is the combination of the cationic polymerisation of Cl_3_PNSiMe_3_ (Fig. 9) with a second block using a phosphoranimine of the type ClR_2_P=N-SiMe_3_, resulting in one “tunable” block of [NPCl_2_]_*n*_, with its wide array of possible substituents, followed by a polyalky/arylphosphazene block.^26^

**Fig. 10:**
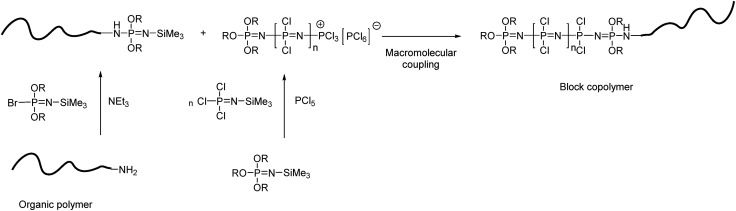
Representative synthetic route to polyphosphazene block copolymers with organic polymers.

Block copolymers combining polyphosphazene blocks with blocks from a second polymer can also be synthesised, for example, the organometallic–inorganic block copolymer poly(ferrocenylsilane-*b*-polyphosphazene),^22^ prepared *via* end group functionalisation of PFS with diphenylphosphine groups which could then act as a macroinitiator for the cationic polymerisation of Cl_3_PNSiMe_3_. Furthermore, a number of block copolymers with organic polymers have been reported using phosphoranimines as capping agents to modify an organic polymer which can then undergo macromolecular coupling with a “living” poly(dichloro)phosphazene (Fig. 10). An example includes the capping of amine functionalised polylactides with bromophosphoranimine,^27^ however, the same synthesis principle can and has been applied to a wide range of organic polymers.

## 3. Polyphosphazenes with advanced architectures

### 3.1 Graft polymers with organic side-chains

The use of controlled polymerisation techniques allows the synthesis of polymers with defined chain length, composition and more complex architectures based on polyphosphazenes. In this regard, the living condensation method described in the previous section can also be used to end-cap polyphosphazenes with polymerizable groups that can then be used to grow organic polymer chains, also described as telechelic polymers. An example here includes the capping with norbornenyl functionalised phosphoranimines which can undergo ring-opening metathesis polymerizations^28^ in essence polynorbornenes with grafted phosphosphazene pendent side arms.

**Fig. 11:**
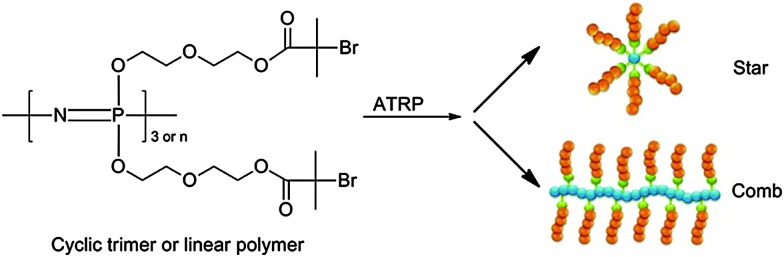
Synthesis of grafted star-like and comb-shaped molecular brush hybrid poly(organo)phosphazenes *via* ATRP. Reprinted with permission from X. Liu *et al.*, *Macromolecules*, 2012, **45**(3), 1417. Copyright 2015 American Chemical Society.^29^

The opposite procedure, *i.e.* grafting of organic polymers onto [NPCl_2_]_*n*_, with its two functional groups per repeat unit, allows relatively simple access to highly dense molecular brush-type polymers.^12^ Alternatively, it is possible to functionalise the backbone with macroinitiators for a ‘grafting from’ approach, as exemplified by the functionalisation with alkyl bromides capable of initiating vinyl polymerisations emanating from the inorganic backbone *via* atom transfer radical polymerisation (ATRP) (Fig. 11). This method was used to prepare a series of densely grafted molecular brushes composed of polystyrene, poly(*tert*-butyl acrylate), and poly(*N*-isopropylacrylamide).^29^ Conversely, the cationic polymerisation route allows the grafting of polyphosphazenes onto multifunctional polymers, as was illustrated by the grafting of [PN(OCH_2_CF_3_)_2_]_*n*_ onto phosphoranimine-terminated PAMAM dendrimers, to give dendrimers consisting of an organic core and polyphosphazenes grafted onto the end of each arm.^8^

### 3.2 Polyphosphazenes with a branched polyphosphazene backbone

**Fig. 12:**
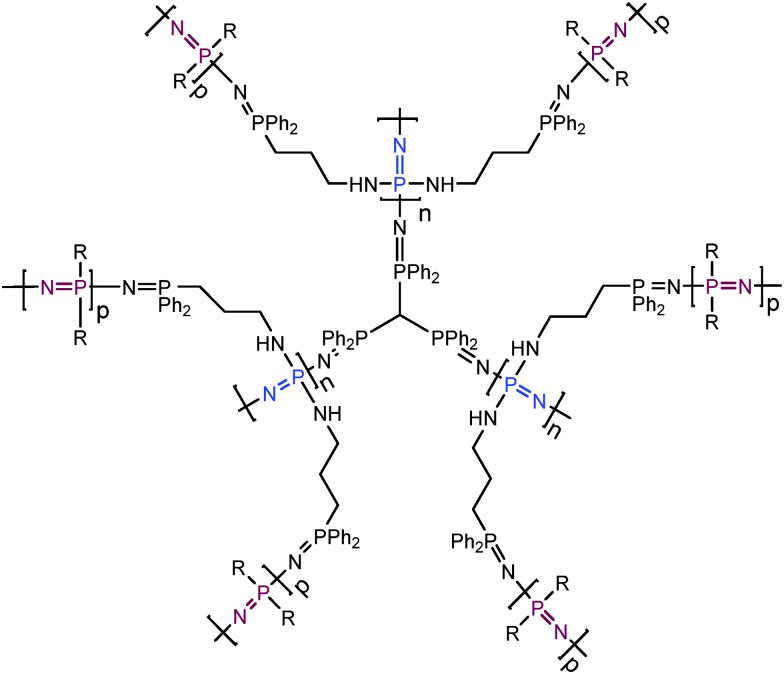
Chemical structure of a polyphosphazene-based dendritic molecular brush, consisting of a star polyphosphazene (generation 1, ‘n’, shown in blue) which was used as a macroinitiator to grow polyphosphazene arms (‘graft from’, generation 2, ‘p’, shown in purple).

Previously reported examples of ‘branched or dendritic’ polyphosphazenes are in fact based on organic polymers, with polyphosphazenes grafted to or from the branched unit. However, a synthetic route to prepare polymers with a polyphosphazene-based branched structure has also recently been developed (Fig. 12).^30^ This method utilises star-like polyphosphazenes (generation 1) substituted with diphenylphosphine moieties. These can act as macroinitiators for the simultaneous polymerisation of a second generation of many arms to produce highly branched polyphosphazenes (dendritic molecular brushes) with high degrees of polymerisation but relatively narrow molecular weight distributions.

### 3.3 Supramolecular structures from polyphosphazenes

**Fig. 13:**
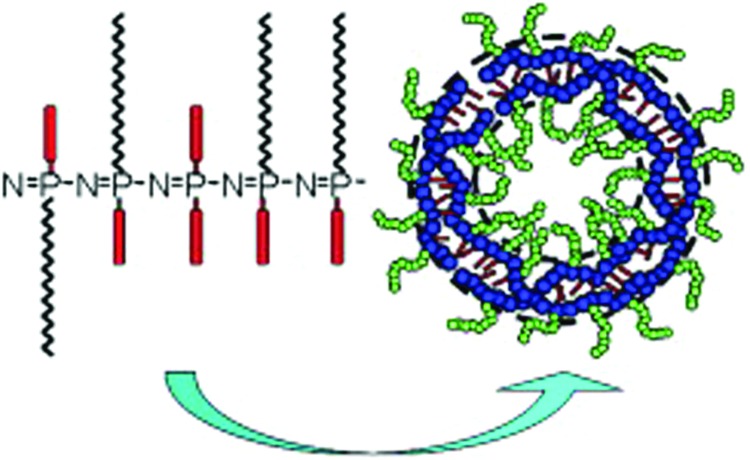
Schematic illustration of amphiphilic poly(organo)phosphazenes with the ability to form micellar and polymersome-like aggregates. Reproduced with permission from C. Zheng *et al.*, *Polymer*, 2009, **50**(5), 1173. Copyright 2009 Elsevier.^31^

The formation of supramolecular structures, for example, polymer micelles and polymersomes, have generated significant interest in recent years. Although commonly made from amphiphilic block copolymers, it is possible to prepare micelles and polymersomes from randomly substituted poly(organo)phosphazenes.^31^ The ability of poly(organo)phosphazenes to form such stable aggregates is presumably due to the conformational flexibility of the polyphosphazene backbone, allowing aggregation of the hydrophobic moieties (Fig. 13). The high functionality of the poly(organo)phosphazenes leads inherently to a wide scope of supramolecular structures with tailored surface properties. One recent example includes poly(organo)phosphazenes with alkene/alkyne organic substituents for the subsequent preparation of highly functionalised polymers *via* thiol–ene/yne addition chemistry.^32^ In this example, the polymers were then decorated with glycosyl moieties to synthesise amphiphilic poly(organo)phosphazenes, which upon self-assembly could undergo specific interactions *via* their surface ligands.

**Fig. 14:**
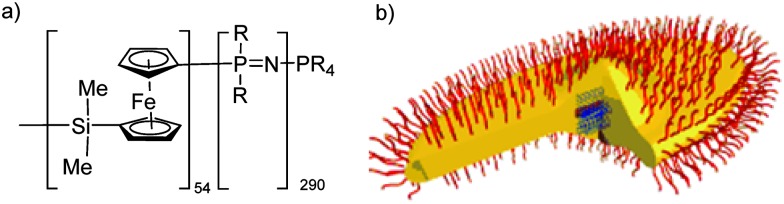
Chemical structure of the block copolymer consisting of poly(ferrocenylsilane) and polyphosphazene (R = OCH_2_CF_3_) blocks (a) and an idealized graphic representation of the pointed-oval-shaped micelles after crystallization-driven living self-assembly (b). Adapted with permission from A. Presa Soto *et al.*, *Angew. Chem., Int. Ed.*, 2010, **49**, 8220. Copyright 2010 Wiley-VCH.^33^

The recently developed methods for the controlled growth of polyphosphazene chains in one-direction opens many opportunities in terms of the preparation of tailored advanced nanomaterials. A prime example here is reported by Soto *et al.* ^33^ who utilize the crystallization-driven living self-assembly of poly(ferrocenylsilane) (PFS) in combination with flexible polyphosphazenes, leading to the formation of stable pointed-oval shaped micelles.^33^ The idealized graphic representation (Fig. 14) shows polyphosphazene coronas in red and the body of the PFS oval in yellow. The centre consists of cylindrical micelles of PFS_34_–P2VP_272_ (P2VP = poly(2-vinylpyridine)) that were used as seed micelles to initiate the self-assembly into this unusual hierarchical structure.

**Fig. 15:**
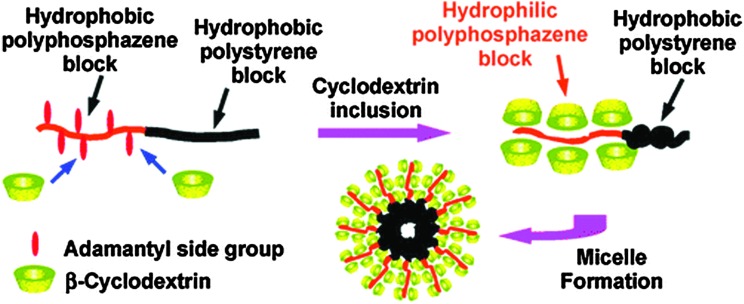
*In situ* formation of amphiphilic polystyrene–polyphosphazene diblock copolymers *via* host–guest inclusion complexes with β-cyclodextrin can be used to prepare supramolecular aggregates. Reprinted with permission from S. Y. Cho and H. R. Allcock, *Macromolecules*, 2009, **42**, 4484. Copyright 2009, American Chemical Society.^34^

The ability to grow block copolymers from polyphosphazenes clearly opens many avenues for macromolecular engineering. Another interesting example here includes the preparation of polyphosphazene block copolymers which can self-assemble to supramolecular aggregates *via* host–guest inclusion complexes with cyclodextrin (Fig. 15).^34^ In this example [NPCl_2_]_*n*_ chains were grown on an amine-capped polystyrene, which upon substitution of the [NPCl_2_]_*n*_ with adamantyl groups, gave a hydrophobic diblock copolymer. The adamantyl group is known to undergo efficient host–guest complexation with cyclodextrin (β-CD) and it was shown that this can also be achieved on the polyphosphazene backbone. Upon mixing with aqueous solutions of β-CD, the block copolymers could be solubilized to micelles at relatively low critical micelle concentrations due to complexation of the adamantyl groups increasing the hydrophilicity of the polyphosphazene block and effectively forming an amphiphilic block. Although themselves not biodegradable, this system represents a proof of principle towards the development of fully degradable micelles if it can be extended to degradable polyphosphazene blocks and replacement of the polystyrene with a bioerodible polymer.

### 3.4 Helical structures with polyphosphazenes

**Fig. 16:**
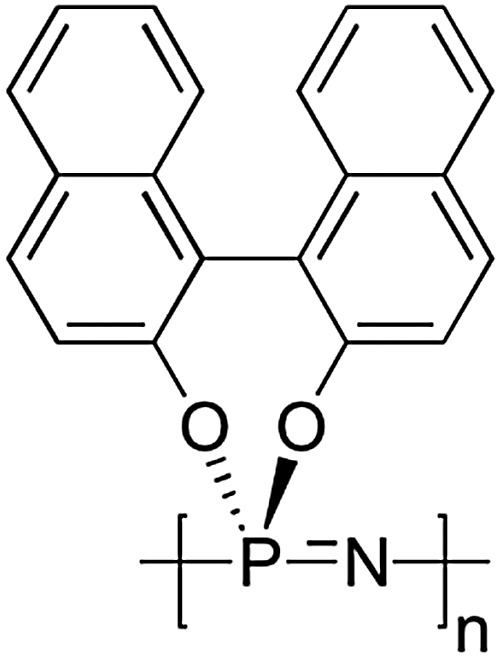
Chemical structure of the chiral poly(2,2′-dioxy-1,1′-binaphthyl)phosphazenes forming a helical conformation.

Helical architectures are well-known and essential structures in nature, such as in DNA and α-helical motives in proteins, hence it is unsurprising that there is much interest in recreating helical structures in synthetic polymers. For this purpose, high molecular weight, spirocyclic polyphosphazenes have been synthesised *via* macromolecular substitution of [NPCl_2_]_*n*_ with binaphthoxy moieties to yield the chiral poly(2,2′-dioxy-1,1′-binaphthyl)phosphazene (Fig. 16)^35^ showing a preferred helical sense in solution and solid state. This polymer is itself intriguing, as the enforced geometry allows complete chlorine replacement of the macromolecular precursor without cross-linking that might be expected from the reaction of [NPCl_2_]_*n*_ with a diol. The glass transition temperature *T*_g_ (∼330 °C) is extraordinarily high for a polyphosphazene which is attributed to the hindered rotation conferred from the binaphthyl substituents. This hindered bond rotation, or atropisomerism, results in polymers which, although behaving as random coils, can form helix-type sequences and short helices in sections of the polymer chain. Through the preparation of block copolymers with [NPMePh]_*n*_, it has also been shown that the helical conformation of the chiral block can induce a preferential helical conformation in the non-chiral block^35^ and that chirality of the helical centre can be transferred upon self-assembly to prepare nanostructures with helical morphologies.^26^

**Fig. 17:**
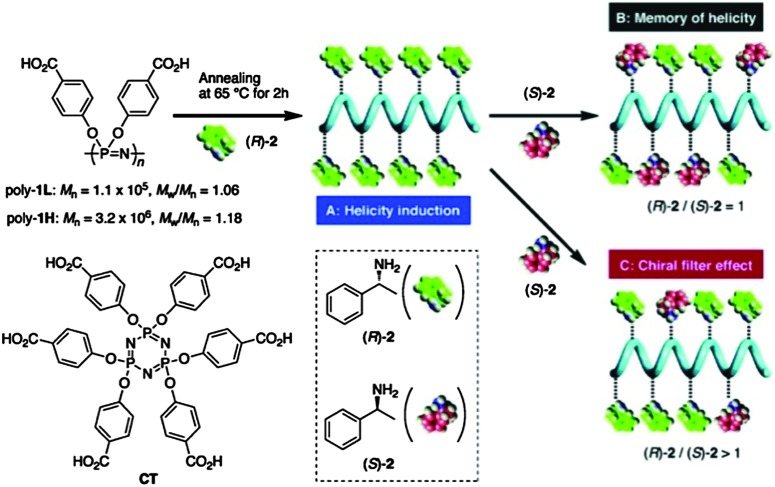
Dynamic helicity of poly[di(4-carboxyphenoxy)phosphazene] and its application as a chirality filter. Note: the polymer referred to as Poly1L here is essentially PCPP, a well-known polyphosphazene investigated for use in medical applications. Reprinted with permission from K. Maeda *et al.*, *Macromolecules*, 2011, **44**, 2457. Copyright 2015 American Chemical Society.^36^

Poly[di(4-carboxyphenoxy)phosphazene] (PCPP) (Fig. 17) is a flexible achiral polymer and is as such optically inactive. Remarkably, however, PCPP has been shown to have a large optical rotation upon complexation with optically active (*R*)- or (*S*)-1-phenylethylamine, transforming into a prevailing one-handed helical conformation (see ref. 36 and references therein), thus acting as dynamic helical polymers. The feature of the dynamic helicity is presumably a function of the high flexibility of the unique polyphosphazene backbone facilitating conformational alignment. These polymers are proposed for numerous applications as chiral materials, for example the use as “chiral filters”^36^ for enantioselective adsorption of racemic amines (Fig. 17).

## 4. Hexachlorocyclotriphosphazene as a building block

A significant amount of literature is dedicated to the preparation of novel materials based on the cyclic phosphazene trimer hexachlorocyclotriphosphazene [NPCl_2_]_3_ (sometimes referred to as HCCP) and the preparation of high molecular weight materials through coupling the rings with multifunctional organic nucleophiles. The ease of accessibility of the cyclic [NPCl_2_]_3_ makes this a particularly attractive preparation route to include the phosphazene unit into polymers. Covalent linkage of the cyclic [NPCl_2_]_3_ can be used to prepare cyclolinear or cyclomatrix, as well as dendritic structures. Cyclolinear structures require only two reactive sites from the six possible in the cyclic [NPCl_2_]_3_ unit, which can be a synthetic challenge, in order to avoid cross-linking but nevertheless numerous examples have been prepared.^1^ Reaction of more than two sites leads to cross-linked, so called cyclomatrix materials, which have shown some recent progress and are thus highlighted in the following section.

### 4.1 Cyclomatrix polyphosphazenes

**Fig. 18:**
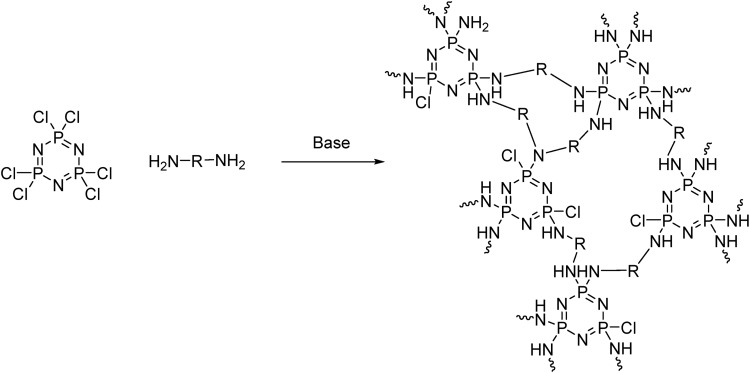
An example synthesis route to cyclomatrix polyphosphazenes using hexachlorocyclotriphosphazene [NPCl_2_]_3_ as a building block.

Precipitation polycondensation, that is the *in situ* formation of an insoluble polymer matrix, can be readily carried out with the cyclic [PNCl_2_]_3_ through nucleophilic substitution with multifunctional organic nucleophiles (Fig. 18). This gives a range of unique functional materials termed ‘cyclomatrix’ polyphosphazenes. Such an approach utilizes the high reactivity of the P–Cl bonds toward nucleophilic substitution, as well as the high number of functional groups. Upon substitution, some chlorine moieties will be left, depending on the reaction conditions. *In situ* processing of the final product is clearly required for such highly cross-linked materials and attention must be paid to the removal of by-products.

**Fig. 19:**
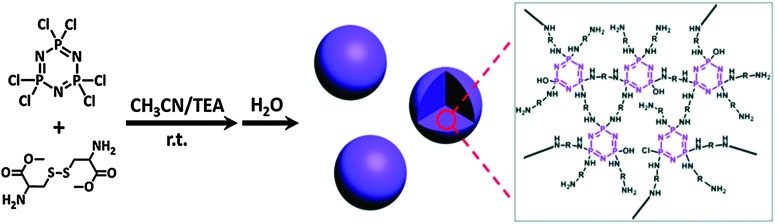
An example of a precipitation condensation reaction to give cyclomatrix polyphosphazenes using [PNCl_2_]_3_ as a building block. Adapted from Z. Huang *et al.*, *Chem. Commun.*, 2015, **51**, 8373 with permission from The Royal Society of Chemistry.^39^

In recent years, clever engineering has evolved the precipitation polycondensation method to prepare cyclomatrix polyphosphazenes with uniform advanced structures such as nano/microspheres and nanotubes.^37^ Both the substitution reaction and the precipitation process influence the chemical structure as well as the size and shape of the products. Most of the chemistry has been developed with aromatic organic moieties whose rigid structures have the required mechanical properties to hold the scaffold together. More recently the approach has also been extended to flexible aliphatic monomers, for example with multifunctional branched polyethylenimines, to prepare more flexible, less rigid materials with further increased functionality.^38^ This brings several advantages including higher functionality and higher degrees of swelling. Recently, Huang *et al.* were able to prepare amino acid ester based phosphazene particles *via* such precipitation condensation chemistry.^39^ The prepolymer/oligomer formation involves the reaction of [PNCl_2_]_3_ with the diamine cystine in acetonitrile (good solvent) followed by particle formation (and curing) upon addition of H_2_O as a poor solvent for the system.^39^ This “water triggered self-assembly” leads to precisely controlled uniform spheres with sizes ranging from 250 nm to 2 μm by adjusting the monomer concentration (Fig. 19).

### 4.2 Dendrimers from hexachlorocyclotriphosphazene

**Fig. 20:**
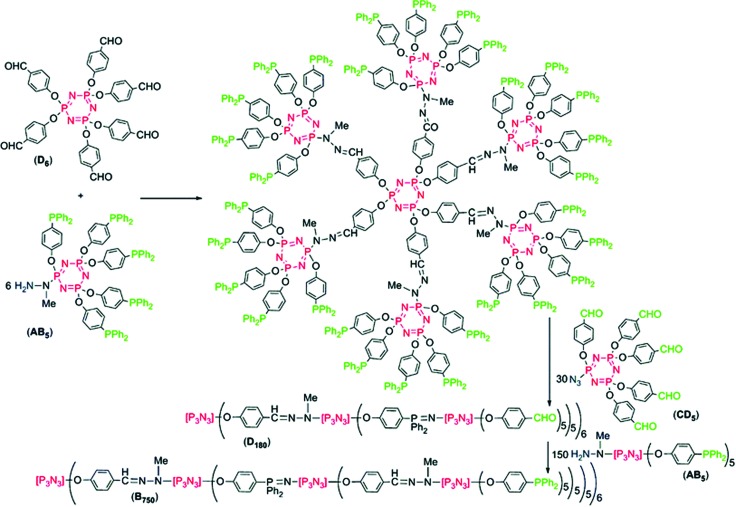
An example of a highly branched dendrimer exploiting the cyclic phosphazene unit to insert a multiplying effect on the branching structure with an AB_5_ type phosphazene based synthon on a hexachlorocyclotriphosphazene core. Reproduced from A.-M. Caminade *et al.*, *Dalton Trans.*, 2016, **45**, 1810 with permission from The Royal Society of Chemistry.^40^

The multiplicity and reactivity of hexachlorocyclotriphosphazene [PNCl_2_]_3_ has also been explored for its effective use as a synthon in dendrimer design, exerting a multiplying effect to the dendrimer due to its six easily functionalized groups. The original approach here was to react [PNCl_2_]_3_ with six diamines, followed by six [PNCl_2_]_3_ molecules and so forth. However, due to the problems of cross-linking and/or high excess of reagents, more elegant approaches have been developed, involving substituted cyclic phosphazenes with orthogonal functionalities. Although many combinations are possible, the synthetically easiest to reach in a controlled manner are the substitution of one chloride with a given functionality, followed by five orthogonal substituents (or *vice versa*) giving AB_5_ type units. These AB_5_ type units give simple access to synthons each offering five branching sites and thus a significant multiplying effect compared to most organic units (which commonly have 2–4 branching sites).^41^ An example here is shown in Fig. 20,^40^ in which two AB_5_ type substituted [PNCl_2_]_3_ moieties are prepared having azide/aldehyde functionalities capable of coupling to a amine/phosphine functionalised AB_5_ monomer in an orthogonal manner. This elegant route allows the preparation of a third generation (*i.e.* three distinct steps) dendrimer with 750 functional end groups. Similar chemistry and the potential of this multiplying effect has been put to great use to prepare a range of well-defined dendritic structures with some properties quite unique from many comparable organic dendrimers. The properties and applications of such phosphazene-based dendrimers have been reviewed recently.^40^

## 5. Formation of bioconjugates with polyphosphazenes

**Fig. 21:**
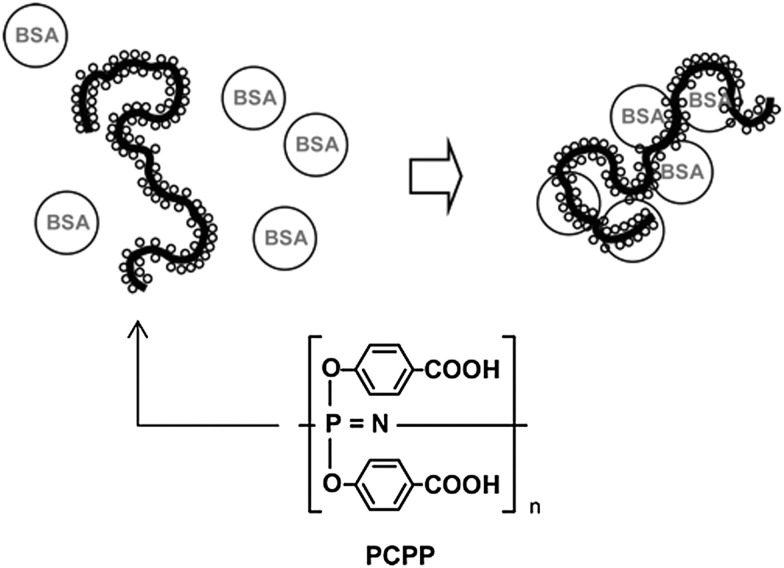
A schematic representation of PCPP, a polyphosphazene-based polyacid, and the proposed conjugation with BSA (bovine serum albumin). Reprinted from A. K. Andrianov *et al.*, *Biomacromolecules*, 2005, **6**, 1375. Copyright 2005 American Chemical Society.^42^

**Fig. 22:**
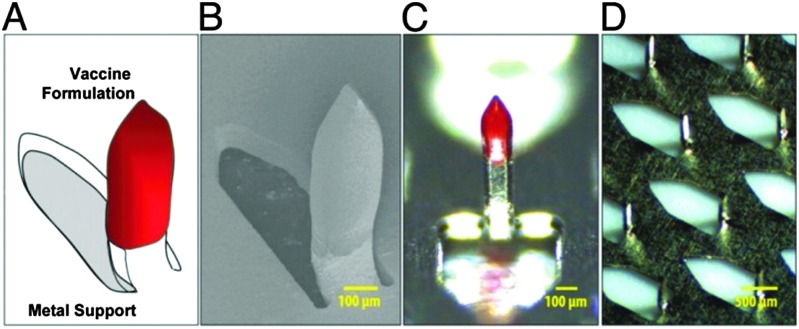
Polyphosphazene coated microneedles for intradermal adjuvant administration: schematic representation (A), scanning electron microscopy image (B), optical microscopy images (C and D). Reproduced with permission from A. K. Andrianov *et al.*, *Proc. Natl. Acad. Sci. U. S. A.*, 2009, **106**(45), 18936.^3^

One of the most outstanding properties of poly(organo)phosphazenes is the ability of its polyacid/polyanionic analogues to bind to biomacromolecules, a property that has wide-ranging potential in biomedicine. A variety of polyphosphazene based polyacids can be prepared. Direct substitution of [NPCl_2_]_*n*_ with the free carboxylic acids is not possible, due to the reaction with the backbone, so alkyl esters are used followed by deprotection under basic conditions. The sodium salt of the polyacids undergo strong non-covalent interactions with biomacromolecules (Fig. 21). Their ability to bind to antigenic proteins has been shown to be far superior to similar anionic polymers based on carbon backbones, due to the high density of binding sites, the higher flexibility of the backbone and the high conformational adaptability this infers.^42^ In works pioneered by A. K. Andrianov and co-workers, the lead compound PCPP poly[di(carboxylatophenoxy)phosphazene] has undergone exhaustive testing into its production,^11^ hydrolytic degradation^43^ and toxicology (p. 54 in ref. 44) and it has been tested with a diverse range of antigens both *in vitro* and *in vivo* in mice, large animals and indeed human clinical trials.^44^ Standard adjuvant formulations based on anionic aluminium salts suffer some disadvantages which could be overcome by using polymer-based adjuvants, not least, for example, for the formation of mechanically stable films. In this regard, it has been shown that PCPP can be formulated into microspheres for drug loading or polymer-coated microneedles for the preparation of skin-patches for facile vaccine administration (Fig. 22).^3^

In a similar vein, cationic polyphosphazenes, usually substituted with quaternized tertiary amino groups, can bind to anionic biomacromolecules^45^ (also reviewed in ref. 6). Investigations of various positively charged polyphosphazenes for gene delivery due to their ability to bind to negatively charged DNA forming polyplexes (sometimes referred to as “nanoparticles” in the literature on the basis of them inherently falling under the ‘nano’ size range of around 100 nm). Interestingly, poly(methylamino)phosphazene is observed to be cationic at physiological pH and its bioconjugation to DNA has been studied in detail.^45^

## 6. *In situ* gelling polyphosphazenes

**Fig. 23:**
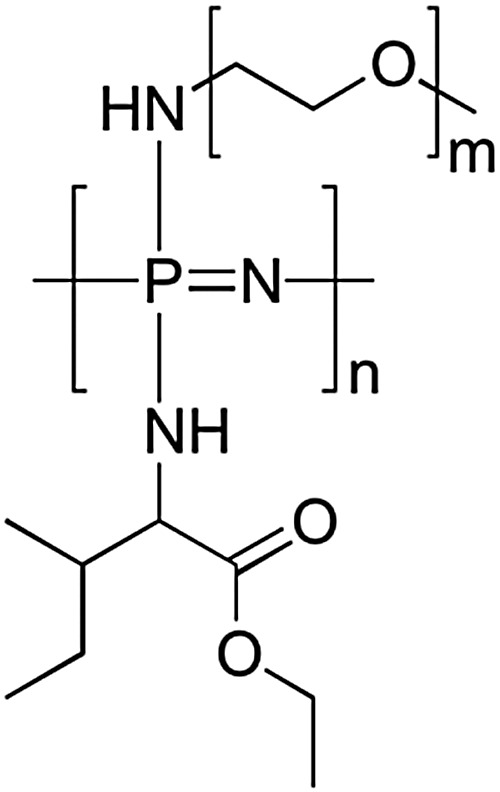
Representative structure of a typical thermosensitive poly(organo)phosphazene. The ratio of the hydrophobic isoleucine ester *versus* the hydrophilic polyethylene glycol units can be used to tailor the solution properties of the polymers. Further co-substitution is used to tune the mechanical and degradation properties.

**Fig. 24:**
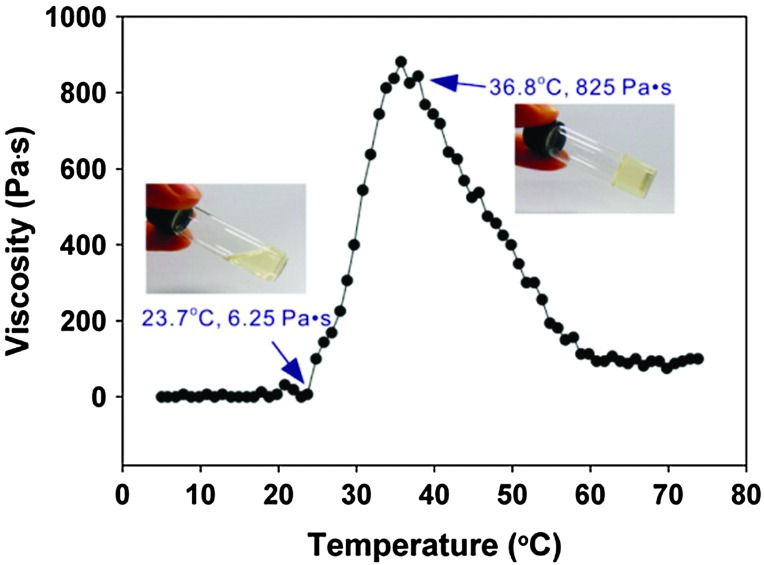
Temperature-dependent sol–gel transition and viscosity change of a suitably functionalised poly(organo)phosphazene. Adapted with permission from Y.-M. Kim *et al.*, *ACS Nano*, 2012, **6**, 5757. Copyright 2015 American Chemical Society.^46^

As discussed earlier (Section 2.4), the macrosubstitution route allows the facile preparation of random block copolymers. One way this synthetic approach can be put to effective use is to prepare amphiphilic polymers which possess a lower critical solution temperature (LCST) transition in aqueous environments. This phenomenon leads to collapse of the polymer chains above the LCST and thus a temperature-triggered agglomeration, followed by precipitation and/or gelation. If the LCST is around or just below body temperature, such triggered phase transitions can be of particular interest for biomedical applications, including drug delivery and tissue engineering. This can be achieved for polyphosphazenes by simple macrosubstitution of the polyphosphazene backbone with organic groups known for their thermoresponsive behaviour, for example, PNIPAm oligomers. Alternatively, it is possible to prepare thermosensitive poly(organo)phosphazenes by co-substitution of the inorganic backbone with hydrophobic and hydrophilic groups. This has been reported in various guises using hydrophobic amino acid esters (most commonly l-isoleucine ethyl ester, co-substituted with hydrophilic PEG oligomers as shown in Fig. 23) in which hydrophobic interactions of the amino acid esters lead to expulsion of H_2_O and thus precipitation and/or gelation above the LCST (Fig. 24).

A large amount of literature has been produced in the last 15 years (mainly by S.-C. Song and co-workers) focussing on the modification and optimization of the mechanical properties and degradation rates of such amphiphilic, thermosensitive poly(organo)phosphazenes, as well as their medical application, in particular the use as injectable hydrogels for drug delivery and theranostics with a wide range of drugs and therapeutic applications being investigated (see ref. 46 and 47 and references therein). Based on variations of the PEG/isoleucine ethyl ester substituted poly(organo)phosphazenes (Fig. 23), further co-substitution of the multivalent [PNCl_2_]_*n*_ can be carried out to tailor the degradation rate and to improve the mechanical stability of such hydrogels, for example, by the addition of photocrosslinkable groups or to covalently bind drugs or biologically active agents. The tunability of the materials, combined with the proven biocompatibility and degradability in combination with the ability to gel rapidly *in situ* at physiological temperatures makes them excellent materials for the use as injectable hydrogels.

**Fig. 25:**
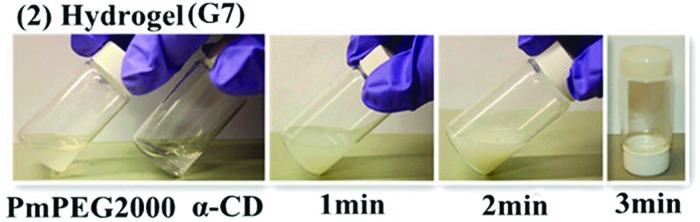
The formation of hydrogels upon mixing through the complexation of poly(organo)phosphazenes containing side oligo(ethylene glycol) methyl ethers (mPEGs) (called here PmPEG2000) with α-cyclodextrins (α-CD). Combination of the polymer with (α-CD) results in rapid hydrogel formation *via* reversible supramolecular assembly. Adapted with permission from Z. Tian *et al.*, *Macromolecules*, 2013, **46**, 2715. Copyright 2015 American Chemical Society.^48^

Supramolecular assembly can also be used to prepare *in situ* gelling materials, for example, polyethylene glycols are well known to form polyrotaxanes with cyclodextrins which tend to undergo physical cross-linking and thus gelation *via* hydrogen-bond interactions. Attaching many short chain, polyethylene glycol monomethyl ether oligomers onto a polyphosphazene backbone recreates this effect (Fig. 25)^48^ and has the advantage to be able to adjust degradation rates thus making them viable materials for biomedical applications, for example in injectable drug delivery.

## 7. Preparation of degradable poly(organo)phosphazenes

**Fig. 26:**
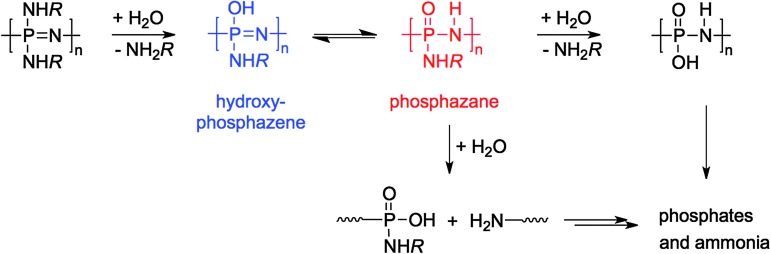
The proposed mechanism for the hydrolytic degradation of poly(organo)phosphazenes.

Much research and many proposed applications of polyphosphazenes, in particular in the biomedical field, are based on their degradability. As with many chemical properties of poly(organo)phosphazenes, the rates of degradation are determined by the nature of the organic substituents and can occupy a full spectrum from biostable, *i.e.* showing no signs of degradation over many years, as is the case for polyphosphazenes with fluoroalkoxy substituents, ranging to, for example, glyceryl derivatives that show complete degradation within several days.^7^ Thus when preparing new poly(organo)phosphazenes it is important to consider and/or where possible to predict what effect the substitution will have on the subsequent degradation rates. Degradation of poly(organo)phosphazenes occurs *via* substitution of the organic side groups with H_2_O, leading to the instable hydroxyphosphazene and phosphazane species, which subsequently leads to chain cleavage and degradation (shown for [PN(NHR)_2_]_*n*_ in Fig. 26). The final degradation products are phosphates and ammonia, both of which can be readily detected by analytical methods,^49^ alongside the given organic substituent. Importantly, the degradation mixture of phosphates and ammonia is widely reported to be benign^7,49^ thus when care is taken with the choice of the organic component, it is possible to prepare materials with non-toxic degradation products. For some biomedical applications, a key advantage of polyphosphazene-based materials is indeed the reported near-neutral, pH-buffered degradation mixture of phosphates and ammonia,^5^ in contrast to the acidic products of the widely used poly(α-esters),^50^ that can be problematic for many uses.

Rules governing the degradation rates of poly(organo)phosphazenes are sometimes complicated by chemical functionality in the organic side groups, but in general it can be said that hydrolysis is observed to be faster for most organic substituents with P–NH–R attachments than P–O–R, due to the strength of the P–O bond. Furthermore, hydrophobic substituents and bulky groups, which can sterically hinder H_2_O attack of the P atom, will tend to deter hydrolysis. Most frequently amino acid esters and dipeptide substituents have been used to prepare degradable poly(organo)phosphazenes,^7^ owing to their biocompatibility and wide availability making it relatively simple to prepare libraries of compounds with varying rates of hydrolysis. The rate of degradation of amino acid ester substituted polyphosphazenes tends to be deferred by larger, hydrophobic groups in the α-position of the amino acid ethyl ester, which can sterically hinder H_2_O attack.

**Fig. 27:**
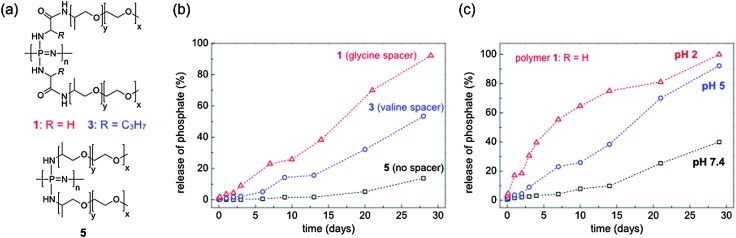
Structures (a) and degradation profiles (phosphate assay) of some exemplary water-soluble, degradable poly(organo)phosphazenes, exemplifying the accelerating effect of hydrolysis by subtle changes in chemical structure (b) and at differing pH values (c). Relative degradation rates are measured by determination of the phosphate backbone degradation product. Adapted from S. Wilfert *et al.*, *J. Polym. Sci., Part A: Polym. Chem.*, 2014, **52**, 287. Copyright retained by authors.^49^

Protonation of the backbone enhances the nucleophilic attack of H_2_O, thus lower pH values (<pH 7) increase degradation rates (Fig. 27c), an observation made for many different poly(organo)phosphazenes,^43,49^ whilst slightly basic conditions are generally observed to have little effect. In this context, poly[di(4-carboxyphenoxy)phosphazene] (PCPP) represents an interesting polymer and is well investigated due to its usefulness in drug delivery. Despite the phenoxy linkage (which would generally be expected to be quite hydrolysis resistant), PCPP can degrade in aqueous environments in medically relevant time-frames due to intramolecular acid catalysis from the carboxyl group.^43^

**Fig. 28:**
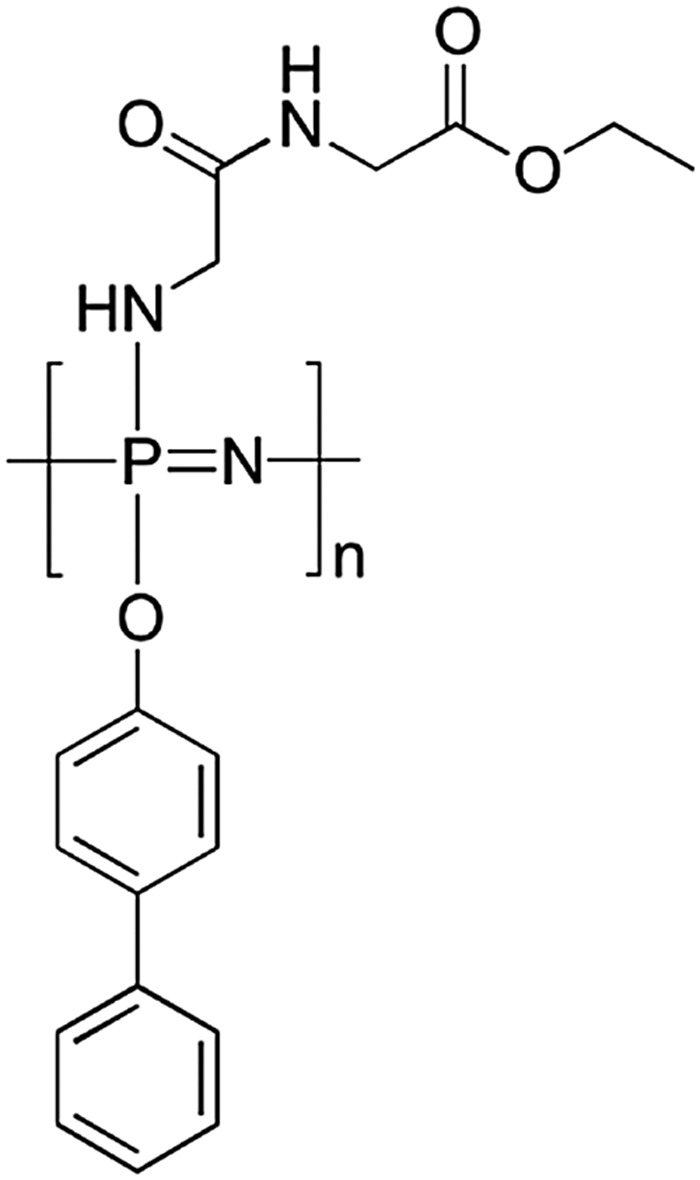
Chemical structure of poly[(glycine ethyl glycinato)(phenyl phenoxy)phosphazene] designed for use as a degradable scaffold for tissue engineering.

Although macrosubstitution with amino acid esters and peptides enhances the rate of hydrolytic degradation, the mechanical properties of such polyphosphazenes are insufficient for some applications. Mixed post-polymerisation macrosubstitution of [NPCl_2_]_*n*_ offers the opportunity here to balance out the properties required in terms of degradation rate and mechanical properties. Poly[(glycine ethyl glycinato)(phenyl phenoxy)phosphazene], a prevalent example here, is shown in Fig. 28, in which the phenyl phenoxy groups deter the rate of hydrolysis but increase the chain rigidity and thus mechanical modulus and *T*_g_ of the polymer. A balance is thus sought between the two functional groups to give the required mechanical properties whilst maintaining suitable degradation rates. This material was designed as a polyphosphazene scaffold for tissue regeneration, that is a biocompatible polymeric scaffold which can support and guide cell growth. Whilst particular mechanical properties are required to function as a scaffold, bearing significant weights to hold the growing tissue, the polymer must also be degradable (to non-toxic products) under physiological conditions in a suitable time-frame so that the material can be replaced by the growing cells.^50^ The synthetic flexibility of polyphosphazenes makes them an ideal platform for tuning the mechanical, chemical and biological properties of tissue engineering scaffolds and has been recently reviewed.^5^

Whilst amino acid esters lead to hydrophobic polymers, water soluble and hydrolytically degradable polymers are also of importance for many biomedical applications due to concerns of the effects of dissolved biopersistent polymers in the body and in the environment. One simple way to achieve this, as demonstrated by Wilfert *et al.*,^49^ is the insertion of amino acid moieties between the backbone and a water-soluble organic substituent Jeffamine (an amine-capped polyalkylene oxide copolymer). These Jeffamine functionalised polyphosphazenes bearing either glycine or valine linkers showed enhanced degradation rates compared to the polyphosphazene without amino acid units (Fig. 27a), with the choice of amino acid ester determining the rate of degradation in aqueous media (Fig. 27b). Such polymers, which can degrade in a controlled manner in clinically relevant time-frames under physiological conditions, are of growing importance for biomedical materials science and in pharmaceutical applications (see ref. 50 for a comprehensive review of the positioning of polyphosphazenes in this rapidly expanding field).

## 8. Conclusions

Overall, poly(organo)phosphazenes represent an extremely versatile class of hybrid inorganic–organic polymers with very differing chemical and physical properties thus leading to their wide range of applications. Although the breadth of properties observed can be bewildering, it is a small number of unique combinations of properties which make polyphosphazenes extremely interesting materials. For instance, degradability for polymers which can be synthesised in a controlled manner is often not met, the combination of chirality and flexibility allowing dynamic helicity, or degradable polymers showing thermosensitive behaviour, to name a few examples encountered in this review. It would appear that it is these unique property combinations, not present in organic polymers, where polyphosphazene-based materials could prove most valuable. The methods and tools to design and synthesise polyphosphazenes with defined properties, as described herein, should enable the preparation of yet further innovative functional materials. Furthermore, recent developments in polyphosphazene synthesis have paved the way for the preparation of advanced architectures and supramolecular structures opening the window to yet more exciting, novel applications.
